# From Nanoparticles to Gels: A Breakthrough in Art Conservation Science

**DOI:** 10.1021/acs.langmuir.3c01324

**Published:** 2023-07-24

**Authors:** David Chelazzi, Piero Baglioni

**Affiliations:** † Department of Chemistry “Ugo Schiff” and CSGI, University of Florence, Via della Lastruccia 3, 50019 Sesto Fiorentino, Italy; ‡ CSGI, University of Florence, Via della Lastruccia 3, 50019 Sesto Fiorentino, Italy

## Abstract

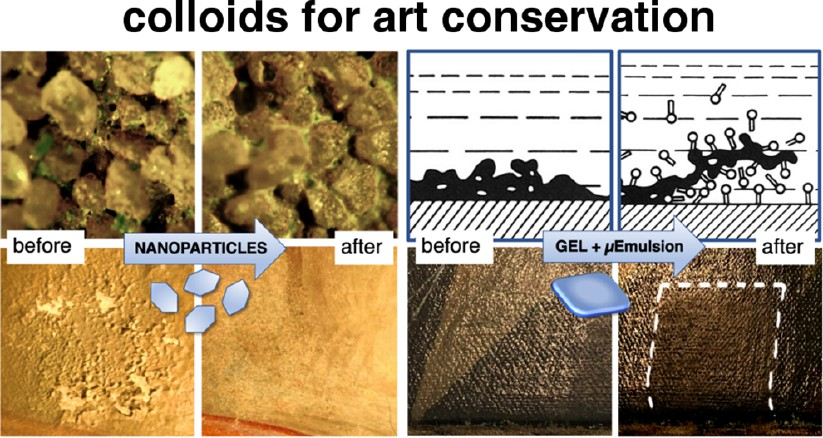

Cultural heritage is a crucial resource to increase our society’s resilience. However, degradation processes, enhanced by environmental and anthropic risks, inevitably affect works of art, hindering their accessibility and socioeconomic value. In response, interfacial and colloidal chemistry has proposed valuable solutions over the past decades, overcoming the limitations of traditional restoration materials and granting cost- and time-effective remedial conservation of the endangered artifacts. Ranging from inorganic nanoparticles to hybrid composites and soft condensed matter (gels, microemulsions), a wide palette of colloidal systems has been made available to conservators worldwide, targeting the consolidation, cleaning, and protection of works of art. The effectiveness and versatility of the proposed solutions allow the safe and effective treatment of masterpieces belonging to different cultural and artistic productions, spanning from classic ages to the Renaissance and modern/contemporary art. Despite these advancements, the formulation of materials for the preservation of cultural heritage is still an open, exciting field, where recent requirements include coping with the imperatives of the Green Deal to foster the production of sustainable, low-toxicity, and environmentally friendly systems. This review gives a critical overview starting from pioneering works up to the latest advancements in colloidal systems for art conservation, a challenging topic where effective solutions can be transversal to multiple sectors even beyond cultural heritage preservation, from the pharmaceutical and food industry, to cosmetics, tissue engineering, and detergency.

## Introduction

Cultural heritage is a fundamental resource: it promotes social inclusion and welfare, fostering job creation through the tourism industry and art market, and carrying historical, aesthetical, and ethical content through generations.^1^ These are crucial advantages to improve our society’s resilience against current and upcoming socioeconomic crises and challenges worsened by climate changes.^1^ At the core of these benefits are the actual works of art, historical sites, and diverse items that need to be maintained and made accessible to implement their positive impact. This is, however, no easy task, as the preservation of cultural heritage assets is continuously endangered by environmental and anthropic risks, including physicochemical degradation by temperature, light, water, and erosion, as well as environmental pollution, microorganisms, natural disasters, vandalism, and even poor restoration interventions with materials that show detrimental effects on the artifacts in the short or long term. In addition, the complex composition and sensitivity of numerous classes of objects, along with the large number of items in actual or potential need of conservation in museums, collections, and sites worldwide, make art conservation an open and challenging topic that calls for urgent solutions. Unfortunately, traditional restoration materials and methodologies often exhibit severe limitations, such as the lack of physicochemical compatibility with the original artifacts’ components, poor spatial or time control in applications that involve time-consuming steps to avoid risks to the artifacts, and the use of hazardous or nonenvironmentally friendly chemicals.^2−4^

In response to these issues, interfacial and colloidal chemistry has taken the lead over the past decades in devising and establishing novel advanced solutions for the remedial and preventive conservation of cultural heritage.^2,3,5−11^ Systems like nanoparticles, composite nanomaterials, and soft condensed matter have proven to be valuable tools in the consolidation, cleaning, and protection of works of art belonging to different artistic and historical productions. While this retrospectively comes as a natural result considering that degradation processes often start at the nano- and mesoscale of the artifacts’ surface and interfaces, it must be noticed that the formation of a scientific framework fully dedicated to the design of colloidal systems for art conservation took some time to emerge, initially starting as germinal ideas not yet validated by common views in science. The first pioneering works were published in *Langmuir* at the beginning of the 2000s,^12−16^ marking a breakthrough in conservation science that, until that point, had been mostly dedicated to the development and use of diagnostic techniques to characterize the composition and degradation processes of works of art.^17−31^ Ten years later, a feature article, also published in *Langmuir* in 2013,^2^ summarized the main advancements and perspectives over two decades of colloids and materials science for the conservation of cultural heritage, showing the potential of colloid science in remedial conservation, which added to its use in understanding degradation processes and devising advanced diagnostic techniques.^32−35^ Nowadays, the field is flourishing with solutions and concepts with large impact even beyond art conservation, potentially interesting transversal fields like food and pharmaceutical chemistry, drug-delivery, detergency, tissue engineering, and others. New challenges have involved the preservation of modern/contemporary art, which has particularly demanding issues, and the need to develop sustainable solutions according to the imperatives of the Green Deal.^36^ These advancements will be reviewed in the following sections, divided by the main classes of developed colloidal materials and ending with current and future perspectives in this exciting field where, despite outstanding results, much can and must still be achieved.

## Nanoparticles and Hybrid Composites

The consolidation of stone, mortars, and murals has historically been one of the main testing grounds for the first formulations of colloidal materials specifically devised to preserve cultural heritage. The conservation task, in this case, is a recurring issue for buildings, stone, and wall paintings exposed to weathering and pollution. Namely, erosion by wind, disaggregation by salt crystallization or water condensation followed by freeze–thaw cycles in pores/cracks, thermal stress, and attack by microorganisms all concur to produce the powdering, flaking, blistering, or even extensive detachment of surface layers from these works of art.^37,38^ Starting from the 1950s, the traditional approach of conservators to reattach loosing parts was the employment of synthetic polymer adhesives (acrylates, vinyl acetate, epoxy resins), applied either in solutions or aqueous emulsions. Synthetic coatings, adhesives, and protective varnishes were deemed as optimal products and thus widely used, since they are inexpensive, are easy to find and apply, and have excellent adhesive power in the short term. In addition, they make the surface hydrophobic (reducing contact with water) and produce color saturation by interposing layers with an intermediate refractive index between pictorial surfaces and air. However, six decades of field experience have shown that these coatings can be highly detrimental as they cap the natural porosity of stone and mortars, resulting in large pressure in the pores when salts crystallize at the stone/mortar-coating interface, eventually producing enhanced detachment and loss of the artifacts’ surface layers. The coatings also yellow and crack as they age, further jeopardizing the treated surfaces.^2,39−41^

**Figure 1:**
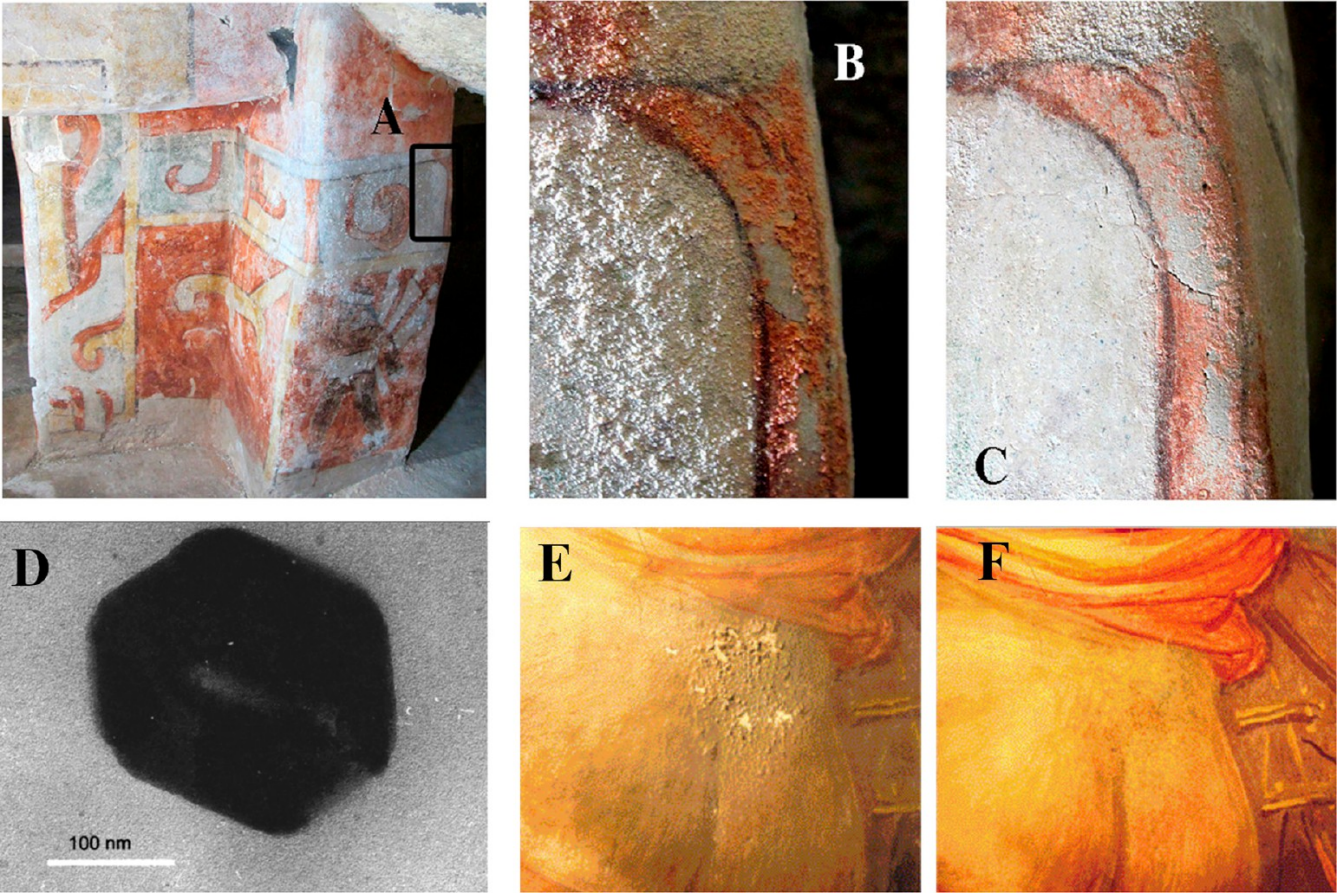
(A) Wall painting belonging to a Mesoamerican archeological site. (B) Details of a flaking surface exhibiting sulfate efflorescence. (C) Same surface obtained after desulfation treatment with ammonium carbonate and application of a mixed calcium and barium hydroxide nanoparticle dispersion. Reproduced with permission from ref (2). Copyright 2013 American Chemical Society. (D) Transmission electron microscopy micrograph of a Ca(OH)_2_ nanoparticle, showing the hexagonal habitus (bar = 100 nm). (E, F) Application of Ca(OH)_2_ nanoparticles dispersed in propanol for the consolidation of the wall paintings by Santi di Tito (16th century) in Florence Dome: (E) before restoration and (F) after restoration. Reproduced with permission from ref (12). Copyright 2001 American Chemical Society.

Coping with these issues, the first paradigm shift from using polymeric adhesives was introduced by Enzo Ferroni in the 1970s, when he devised a method based on solutions of ammonium carbonate and barium hydroxide to extract soluble sulfates from wall paintings and have Ca(OH)_2_ formed *in situ* in the murals’ pores.^2,42^ The newly formed hydroxide reacts with CO_2_ in the atmosphere (carbonation process) to produce layers of calcite that restore cohesion and adhesion in the painted layers, mimicking the setting of a fresco. Noticeably, the method launched a new principle in art restoration where only materials compatible with the originals were employed, minimizing drawbacks in the short and long term. The method was demonstrated extensively by Ferroni and Baglioni, allowing the restoration of frescos from the Italian Renaissance, e.g., in Florence (Italy), where numerous paintings had been severely damaged by the 1966 flood.^2,43^ Working on the method’s limitations, i.e., risks to alkali-sensitive pigments when exposed to the strongly alkaline solutions, provided the chance for the formulation of colloidal dispersions of calcium hydroxide particles in nonaqueous solvents, which, starting from the early 2000s, proved to be safe and valuable alternative solutions to the synthetic polymer coatings.^2,12^ Inspiration for the synthesis of colloidal Ca(OH)_2_ also came from the works of Egon Matijević et al., including seminal works in *Langmuir*, on the syntheses of metal oxide and hydroxide particles by precipitation from sol solutions, taking advantage of temperature, pH, additives, and co-ions.^44−46^ Colloidal Ca(OH)_2_ particles were thus synthesized by Baglioni et al. with different bottom-up or top-down processes and then stably dispersed in short-chain alcohols (ethanol, propanol) without additives, with concentrations in the range of 5–30 g/L.^2,12,47^ The dispersions can be applied by brushing, spraying, or injection, and the particles are carried in the artifacts’ pores by the alcohol solvents, which exhibit good wettability of stone/mortars and balanced evaporation rates. When alcohol evaporates, the particles deposit in the pores and undergo carbonation to rebuild a layer of calcium carbonate that acts as microgrouting and restores the mechanical properties of stone, mortars, and wall paintings. The amount of Ca(OH)_2_ consolidant delivered onto the artifacts, and thus the consolidation effect, is enhanced with respect to the Ferroni method. In addition, switching from aqueous alkaline solutions to dispersions in alcohols allows for the treatment of sensitive iron- and copper-based pigments without alterations. The dispersions were validated on numerous case studies through the decades, restoring heritage assets spanning from the European Renaissance to Mesoamerican Mayan murals (see Figure 1), setting a new benchmark in consolidation methodologies.^5,9,10^ Following these pioneering applications, numerous variations in the synthetic approaches have been proposed by several groups, including the use of organic solvents or surfactants to assist the particles’ precipitation, solvothermal synthetic processes to obtain the particles from metallic calcium, and formulation of mixed hydroxide dispersions to tackle different types of artistic/historical substrates.^47,48^

In parallel to consolidation, dispersions of alkaline earth metals nanoparticles were formulated by Baglioni et al. since the early 2000s to counteract degradation by acids in cellulose-based items such as paper manuscripts and artworks, canvas, or easel paintings, and even wood.^2,13,15,16,49^ Acids catalyze the hydrolysis of cellulose, leading to mechanical failure in the degraded objects, and acidity sources range from gas pollutants hydrated in the cellulose fibers’ moisture, to ancient inks, products, and residues from the papermaking process, paper degradation products, emission from wooden boxes and display cases, and glues or adhesives employed in the restoration practice.^50−53^ Waterlogged wood in shipwrecks can become highly acidic when reduced sulfur compounds, formed by bacteria in polluted waters, permeate the timbers and then oxidize to sulfuric acid after the shipwreck is salvaged. Famous cases are the Vasa and the Mary Rose.^54,55^ In addition, the traditional wood consolidant, polyethylene glycol (PEG), can degrade to formic acid by iron ions that are present in waterlogged wood from the corrosion of nails, bars, bolts, etc.^56^ The hydroxide nanoparticles have positive surface charge and adhere to the negatively charged cellulose fibers; there, they neutralize acidic moisture, and the remaining particles turn into carbonate, forming a harmless alkaline reserve against recurring acidity. In this way, pH can be adjusted around neutral values, discouraging hydrolytic and even oxidative processes and controlling alkalinity, as opposed to the use of aqueous solutions of hydroxides that are traditionally used in mass deacidification.^2,13,15,16,57−60^ The method has been extended and varied over the years to tackle sensitive collagen-based artifacts (leather, parchment), or to neutralize acidic emissions from wooden surfaces.^61,62^

A crucial aspect in the use of alkaline earth metal nanoparticles involves the carbonation process. Temperature and relative humidity deeply affect the process’ kinetics, along with the surface area and size distribution of the particles, the presence of compounds adsorbed on their surface, and the porous structure of the target substrate.^63−65^ Recently, the formation of carbonate on films of Ca(OH)_2_ nanoparticles was evaluated by the Boundary Nucleation and Growth Model (BNGM), quantifying the effects of surface area and temperature that boost the carbonation kinetics along with high relative humidity.^66^ Controlling the rate of the process opens different applications: fast carbonation is advocated when the particles are used to counteract acidity, as calcium carbonate is a milder alkali that is safer on aged, oxidized cellulose fibers. Instead, slower carbonation might be indicated in consolidation cases to foster the formation of larger crystalline carbonate domains and, thus, enhanced cohesion of powdering stone or murals.

**Figure 2:**
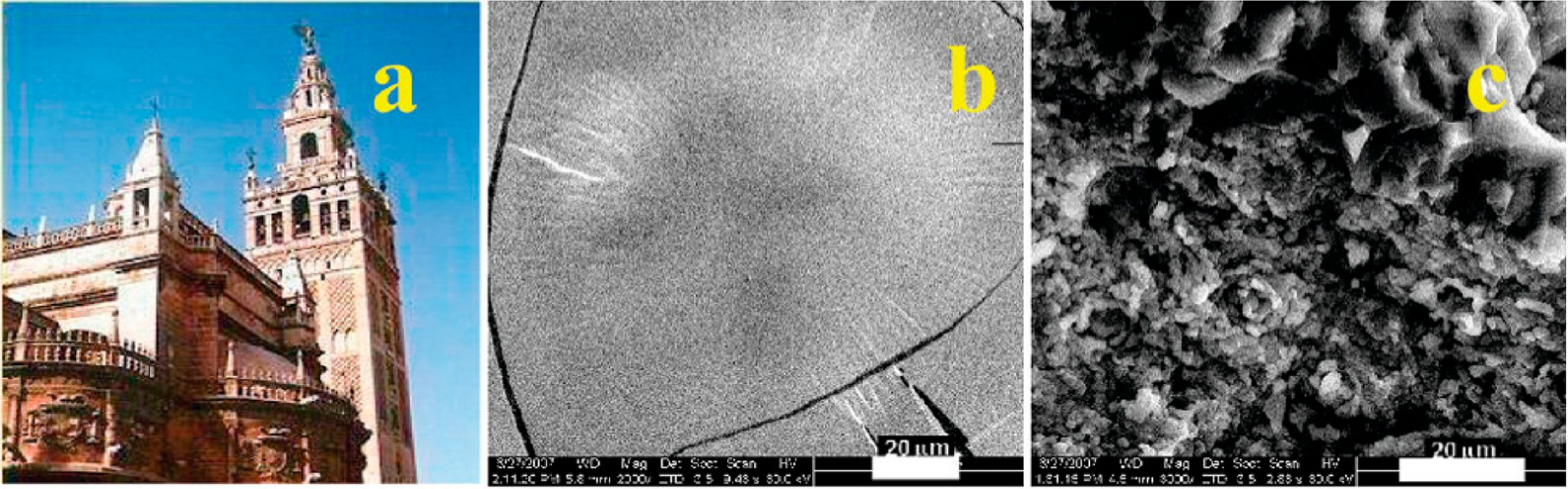
(a) Sevilla cathedral. (b, c) Scanning electron microscopy micrographs of the same biocalcareous stone: (b) consolidated with a commercial consolidant (partially prepolymerized tetraethoxysilane (TEOS), dibutyltin dilaurate catalyst, and ethanol), which forms a dense coating inside the stone, severely affected by cracking (bar is 20 μm), and (c) consolidated with a sol containing TEOS and an amine primary surfactant, yielding a continuous and crack-free mesoporous coating of uniform spheres, where original stone minerals are also observed (bar is 20 μm). Reproduced with permission from ref (72). Copyright 2008 American Chemical Society.

Another fundamental class of colloidal materials largely adopted in stone consolidation is silica and its derivatives to strengthen silicate rock or buildings degraded by erosion and weathering. Commercial coatings based on colloidal silica were already used since the 1970–1980s,^67−69^ and cohesion gain induced on stone by nanosilica was also proved recently,^70^ while the most popular consolidants on the market are traditionally based on alkoxysilanes.^71^ However, the latter form networks in the stone that tend to crack, diminishing their effectiveness. Alternatively, Mosquera et al. developed in the late 2000s a surfactant-assisted sol–gel process to produce a crack-free uniform mesoporous network of silica nanoparticles in the stone pores;^72^ see Figure 2. The particles are formed in nanoreactors created by surfactant micelles, which are mixed with a silica oligomer.^73^ The produced mesoporous structure reduces the capillary pressure during drying, preventing the formation of cracks in the network. The system can also be modified by the addition of polydimethylsiloxane, fluorinated compounds, as well as inorganic nanoparticles (TiO_2_, CuO) to impart superhydrophobicity, photocatalytic or biocidal properties to the network.^74^ In addition to these significant advances, it must be noticed that nanoparticles of Ca(OH)_2_ have also been combined with either alkoxysilane or silica nanoparticles to yield composites that are active in the consolidation or stone or earthen materials.^48,75^ For instance, the composite with silica nanoparticles forms calcium silicate hydrate (CSH) *in situ* in the earthen bricks, providing resistance to abrasion and wet–dry cycles, opening new perspectives in the preservation of earthen construction materials with potential impact both on the preservation of historical sites and the development of sustainable architecture in growing economies. Based on similar colloidal chemistry, other current and future perspectives involve the use of advanced smart materials for the preservation of concrete historical buildings, a significant issue in modern/contemporary heritage conservation.^76^

Colloidal compounds are also being developed for the consolidation of canvas paintings, where the need is to overcome the limitations of synthetic adhesives or natural glues that can alter the optical properties of painted layers or develop detrimental chemicals.^53^ In particular, starch nanoparticles,^77^ fibroin-nanocellulose hybrids,^78,79^ or keratin mixed with halloysite nanotubes^80^ have been recently proved to be promising consolidants or adhesives for paints and textiles, showing the great potential of biopolymers in the conservation of cultural heritage.

Finally, colloids are being increasingly developed and proposed for the protection of works of art, i.e., preventing damage and alterations by either acting on the works’ surfaces (remedial conservation)^81^ or devising tools to neutralize environmental degradation agents before they reach the artifacts (preventive conservation).^9,10^ Among the latest applications, we mention here multifunctional halloysite nanotubes,^82^ the use of graphene veils to prevent the fading of colors,^83^ colloidal semiconductor photocatalyst or nanocrystals for self-cleaning and degradation prevention,^84,85^ soil colloids as templates to protect jade,^86^ antioxidant bionanocomposites or antifouling coatings,^87,88^ sol–gel or nanocarriers to protect bronze from corrosion,^89,90^ mesoporous silica nanoparticles for the controlled release of antimicrobials,^91^ cellulose nanocrystals or lignin nanoparticles as UV absorbers,^92^ and organic–inorganic composites or metal organic frameworks to absorb volatile acids in enclosures.^93−95^ These are all key studies to show the vast potential impact of nanoparticles and nanocomposites in cultural heritage conservation, thanks to properties that surpass those of conventional restoration materials. Current challenges involve scaling up the production of these innovative systems, implementing green synthetic processes, transferring the best products to the conservation market, and linking with transversal sectors that can benefit from the new materials.

## Nanostructured Fluids

A complementary task to consolidation and protection is the cleaning of works of art, i.e., the removal of any undesired layers from the works’ surface, including dirt/soil, contaminants, corrosion, or degradation products, as well as aged varnishes/coatings/adhesives, overpaint, and vandalism. Indeed, cleaning is one of the most recurrent restoration interventions, and the common issue to practically all cases is to achieve selective removal of the undesired layers without affecting the artifacts’ original components.^4,96^ Given the complexity of surfaces and interfaces in works of art, and their frequent sensitiveness to aqueous solutions or solvents, achieving safe, selective, and time-effective cleaning is often a challenging task.^97−100^ Historically, cleaning operations employed organic or biological materials, such as wine, vinegar, or bile fluids, which already contained solvents, surfactants, and colloidal soft matter. However, scientific awareness and rigorous materials design have been replacing serendipity and trial-and-error starting only in the 1980s. The traditional restoration practice is currently based on the use of classic detergency and chemistry of solutions,^96^ polymers,^101^ and surfactants.^3,101^ The basic approach is to match the solubility of soil/coatings with solvent blends using solubility parameters^96^ and then employ swabs or polymeric thickeners (e.g., poly(acrylic acid) (PAA) or cellulose derivatives) to apply the blends,^101^ swelling or dissolving the undesired layers. Conservators are also increasingly adopting aqueous systems such as solutions of surfactants, chelating agents, and enzymes. The use of these aqueous solutions, along with regular oil-in-water (o/w) emulsions and polymeric thickeners, was systematically proposed in the 1990s by Wolbers.^101^ More recently, he also investigated the regulation of pH, conductivity, and ionic strength in aqueous cleaning systems to reduce the swelling or leaching of original components from acrylic painted layers.^102^

However, despite the progress they have introduced over serendipitous approaches, these methodologies exhibit limitations that have required the development of new solutions. Solubility parameters are not exhaustive in describing the interactions between solvents, varnishes, and paintings’ binders,^3^ and the use of nonconfined, or poorly confined, solvent blends has scarce selectivity. As a result, cleaning operations can typically be time-consuming to allow step-by-step checking of potential damage to painted layers. Regular o/w emulsions are only kinetically stable, and their cleaning capacity can be dramatically boosted, along with stability, by switching to microemulsions, where the interfacial area of the nanosized micelles containing the solvent droplets is much larger with fast exchange dynamics.

**Figure 3:**
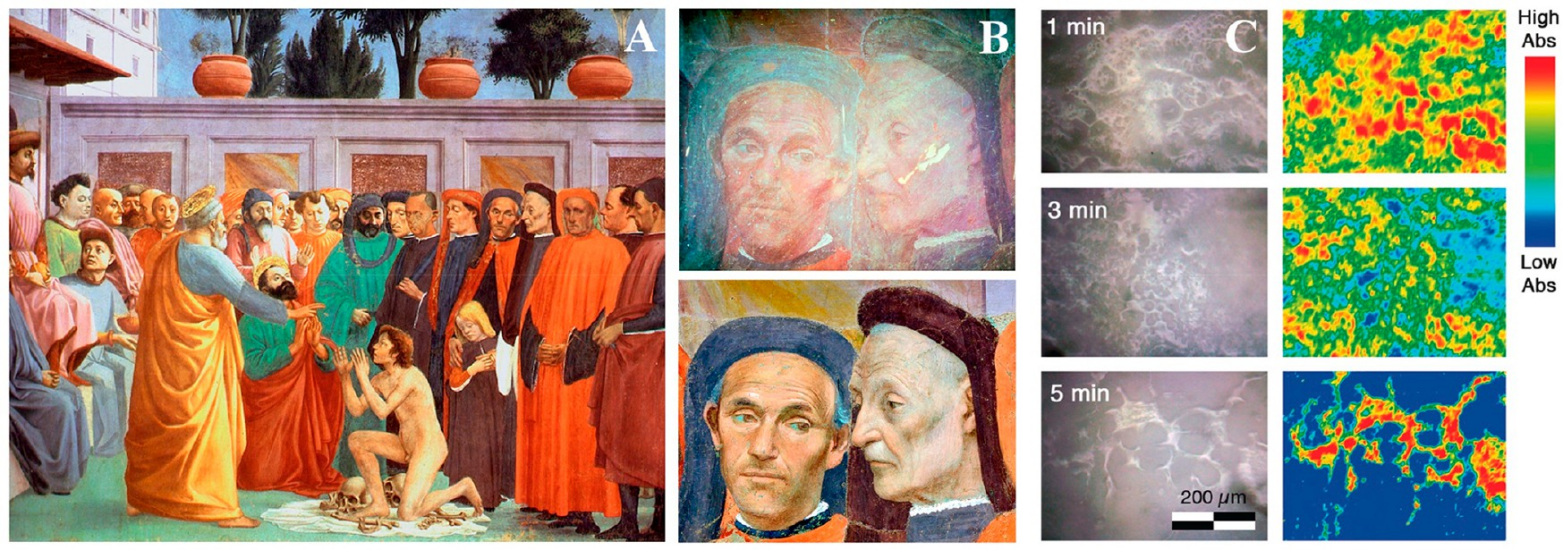
(A, B) Wall paintings by Masaccio and Masolino in the Brancacci Chapel, Florence. (B, top) Wax spots under UV light before cleaning. (B, bottom) Same area under visible light after cleaning with an o/w microemulsion. Reproduced with permission from ref (2). Copyright 2013 American Chemical Society. (C) FTIR 2D imaging of a mortar with a polyacrylate coating, which dewets by action of an aqueous nanostructured cleaning fluid after 1, 3, and 5 min. Each row shows the image of the surface under VIS light (left) and the FTIR map of the polymer peak at 1735 cm^–1^ (C=O stretching; right). Red–yellow pixels indicate high absorbance of the polymer; green–azure = low absorbance; blue = polymer was not detected. Scale bar: 200 μm. The spatial resolution of the chemical maps is 5.5 μm. Reproduced with permission from ref (11). Copyright 2018 Wiley-VCH.

Coping with these issues, an alternative methodology for cleaning works of art was already developed by Ferroni and Baglioni starting from the 1980s, using a different scientific framework based on colloidal physical-chemistry and condensed soft matter.^2^ The new methodology was then continuously implemented to target a growing number of artifact classes in a series of studies, several of which were published in *Langmuir*.^2,103−105^ Namely, taking inspiration from a seminal work by De Gennes and Taupin on the stability of interfacial surfactant films in water/oil/surfactant systems,^106^ in 1986 an o/w microemulsion was specifically designed for the first time to address the removal of wax contaminants from Renaissance frescos in Florence (Italy).^2^ The system was mostly aqueous (ca. 87% w/w) with limited content of solvent (dodecane, 10%), surfactant (ammonium dodecyl sulfate), and cosurfactant (pentanol) and was loaded in a cellulose poultice that adsorbed the wax droplets as they were removed from the fresco by the microemulsion. Inclusion of wax in the nanosized oil droplets and wax detachment driven by osmotic flows (following ionic surfactant adsorption at substrate–wax interfaces) were deemed as the main factors to explain the high efficacy of the microemulsion, which completely removed the contaminants with no alterations to the paint; see Figure 3.^2,11^ These detergency mechanisms also play a fundamental role in the removal of other low molecular weight undesired layers, like greasy soil, aged varnishes based on insect or plant extracts, or particulate soil.^11^

**Figure 4:**
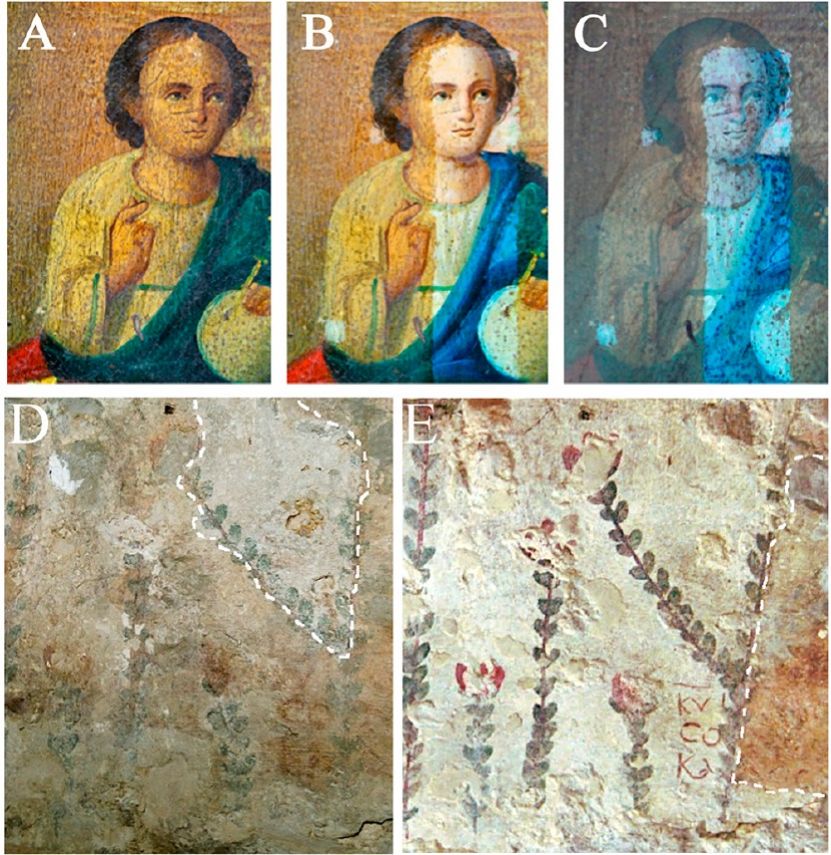
(A–C) Removal of an aged vegetal (terpene-based) varnish from the surface of an oil painting on wood panel. (A) Visible light image of the painting before cleaning. (B, C) Visible light (B) and UV fluorescence (C) image after leaning with a nanostructured cleaning fluid (NCF), which removed the aged varnish. Reproduced with permission from ref (122). Copyright 2018 American Chemical Society. (D, E) Application of an NCF to remove aged synthetic polymer coatings from wall paintings in the Annunciation Basilica in Nazareth (Israel): (D) before cleaning and (E) after cleaning. In the dashed box, an area is highlighted where the polymer coating has been partially removed (D) and left untreated as a reference for the evaluation of the cleaning result (E). Reproduced with permission from ref (105). Copyright 2012 American Chemical Society.

Successively, different nanostructured cleaning fluids (NCFs) were also developed, using nonionic surfactants and partially water-soluble solvents, which are mostly found in the fluid’s continuous aqueous phase and only partially in the surfactant micelles.^3,8−11^ These NCFs were designed to remove aged polymeric coatings (e.g., acrylate, vinyl acetate, epoxy), which they do following nonclassic mechanisms. Essentially, these NCFs promote the dewetting of polymer layers (see Figure 3), driven by two main factors: (1) good solvents and the surfactant swell and mobilize the polymer chains, and (2) surfactant molecules favor the formation of interfaces and polymer detachment areas, lowering the activation energy to initiate dewetting and speeding up its kinetics. Even in cases where the presence of amphiphilic additives in the coating film discourage its dewetting from the artistic substrate, the fast dynamic exchange of solvent, surfactant, and cosurfactant from the NCFs continuous and dispersed phases to the polymer layer causes its swelling, softening, and feasible detachment. Overall, these features are key to achieve the high versatility and efficacy that the NCFs have exhibited in the past decades over a range of case studies spanning from classic frescos and the Renaissance to modern/contemporary masterpieces by Pablo Picasso.^3,5,6,8−11^Figure 4 shows two examples where the NCFs were used to remove aged varnish from a painted wood panel and a complex superimposition of synthetic polymer coatings accumulated on wall paintings in the Annunciation Basilica in Nazareth (Israel) owing to past restorations in the past decades.

In addition, inverse water-in-oil microemulsions have been explored by Ormsby et al., with the rationale of using a hydrocarbon continuous phase to limit possible alterations of paint layers (especially on highly water-sensitive modern/contemporary canvas paintings), as well as an aqueous dispersed phase with chelators to remove hydrophilic soil.^107,108^ Promising results have been obtained, and future improvements will be dedicated to reducing the surfactant content that, in some cases, can consistently exceed 10% (w/w), requiring rinsing steps after the cleaning interventions.

Finally, other promising approaches include the use of hydrophobically modified halloysite tubular nanotubes as reverse micelles for w/o emulsions^109^ or of Pickering emulsions to design nanostructured fluids for cleaning art, for instance, by modifying halloysite nanotubes with surfactants to form micelles able to disperse hydrocarbons in o/w systems.^110^

Current challenges involve the formulation of nanostructured cleaning fluids using “green” solvents (e.g., alkyl carbonates and other esters, food grade oils) and cleavable or biodegradable surfactants.^8,111^ The goal is to keep the same versatility and efficacy of state-of-the-art formulations while adopting sustainable components, and the outcomes of this research are expected to impact also on detergency, cosmetics, and other fundamental industrial fields.^8^

## Gels

As fundamental as they are in many sectors of material science and colloids, gels have also gained growing attention in cultural heritage conservation through the past decades, in particular polymer gels designed and synthesized with different approaches.^2,5,8−11,112−116^ The first main rationale relies on the time and spatial control achievable in art cleaning operations by confinement of cleaning fluids into gel matrices. This allows safe cleaning interventions without the need for lengthy steps checking for possible damages to water- or solvent-sensitive artistic surfaces, overall making the operations time-effective and allowing safe cleanings, in some specific cases ones not achievable with conventional conservation methodologies. In addition, retention in gels confines the interaction of cleaning fluids with soil/coatings to the gel–artifact interface, where the fluids can safely work to swell or detach undesired layers. Osmotic balance and gel tortuosity control the fluid dynamics at the interface, producing effective and tailored cleaning. The design of the gel architecture is thus crucial to maximize these features and overcome the limitations of thickened or nonconfined aqueous solutions or solvent blends. Factors such as the polymers’ chemical composition, hydrophilicity/hydrophobicity, molecular weight, as well as the type of polymeric network and the combination of different compounds all are involved in the formulation of gel matrices tailored for specific cleaning tasks.

Historically, one of the first formulations developed to improve on traditional thickeners was a poly(vinyl alcohol) (PVA)–borate network regulated by the addition of a cosolvent (e.g., short chain alcohols, propylene carbonate, cyclohexanone) that produces structuring of the network.^117^ The type and amount of cosolvent loaded, along with the PVA molecular weight, affects the rheological properties of these systems, which can be made retentive and highly viscoelastic so that they are easily applied and then peeled off the surface in one step after the cleaning interventions. In addition, the presence of the cosolvent widens the solving power of the formulation to target the removal of aged varnishes/coatings of different polarity. Even though the PVA–borate dispersions do not exhibit the rheological behavior of a true gel network, these systems marked a significant step forward from traditional PAA- or cellulose-based thickeners used in the traditional cleaning practice, which are prone to leave polymer/surfactant residues that require potentially invasive rinsing.^118^

True chemical hydrogel networks, instead, were formulated specifically for art cleaning tasks by exploring, respectively, acrylamide/bis(acrylamide) for radical polymerization or semi-interpenetrated poly(2-hydroxyethyl methacrylate)/poly(vinylpyrrolidone) networks (pHEMA/PVP semi-IPNs), in three different studies that appeared in *Langmuir* in the late 2000s to early 2010s.^119−121^ In the first case, the gels were obtained by radical polymerization of the acrylamide monomer and *N*,*N*′-methylene bis(acrylamide) (as cross-linker), yielding a 3D covalent network with tunable porosity. Interestingly, the network was also functionalized with magnetic nanoparticles, which were associated with acrylamide ethylene oxide polymers, producing a nanomagnetic sponge that could be removed from the artifact surface by a small magnetic field, avoiding any mechanical stress during the gel removal step.^119^ In the second case, HEMA was polymerized by radical reaction in the presence of linear PVP, producing a pHEMA chemical network where PVP was entangled.^121^ The advantage in using a semi-IPN is that it exhibits a combination of the best pHEMA and PVP properties, i.e., respectively, excellent mechanical properties and high hydrophilicity. This allowed the formulation of highly viscoelastic porous gel networks with improved retentiveness and thus the safe cleaning of weak, strongly decohered painted surfaces without the risk of removing loose pigments or leaving polymer residues. These hydrogels were loaded with aqueous solutions or even o/w microemulsions, to remove soil and aged coatings/adhesives from canvas, marble, or painted layers, and it was verified that the gels and microemulsions nanostructure was not dramatically altered by their combination, preserving their functionality even in complex cases like the cleaning of watermark paints or the removal of scotch tape adhesives from artworks with sensitive dyes/inks.^122,123^

**Figure 5:**
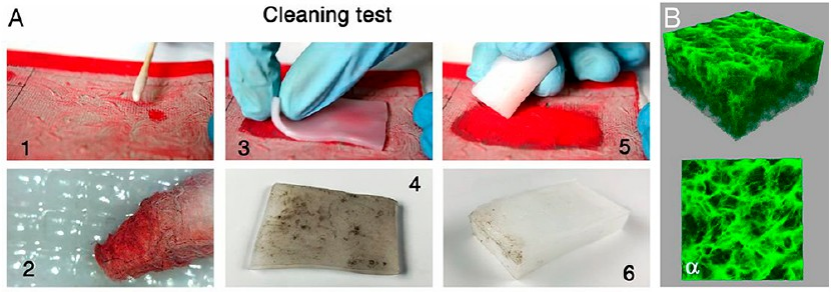
(A) Removal of artificial soil from a water-sensitive oil painting.^1,2^ Removal using a swab soaked with a cleaning aqueous solution; some red pigment is removed along with the soil.^3,4^ Application of a “twin-chain polymer network” (TC-PN) hydrogel sheet on the soiled surface. Only soil, and no red pigment, adheres to the gel sheet following the application.^5,6^ The cleaning is completed using the TC-PN hydrogel shaped as an eraser gum; no red pigment adheres to the eraser gum. (B) Laser scanning confocal microscopy image of the TC-PN network (2D view of the top horizontal planes in panel (A)). Reproduced with permission from ref (124). Copyright 2020 National Academy of Sciences.

PVA was recently used for the formulation of a novel class of gels, the so-called “twin-chain polymer networks” (TC-PNs) (see Figure 5).^124^ The key concept in this case is the use of two types of PVA in the sol mix, differing in their hydrolysis degree and molecular weight. This causes polymers’ demixing in the aqueous environment and the formation of micrometric blobs of a lower molecular weight type (L-PVA) dispersed in the solution of the higher molecular weight polymer (H-PVA). The blobs are elongated upon freezing of the sol, while H-PVA is involved in the gel walls formation. By washing L-PVA, one thus obtains a spongy, disordered, and interconnected porous network, as opposed to ordered stacks of channel-like pores produced by freezing of the sole H-PVA sol. Some of the L-PVA is retained in the wall structure, making it more compliant to mechanical stress. Overall, the characteristic porosity and adhesion properties of the TC-PNs yield homogeneous soil capture and removal even from rough painted surfaces whose cavities are hardly accessible to the pHEMA/PVP or other rigid gels (agar, gellan). These features, along with the possibility of uploading aqueous solutions and o/w microemulsions, have made the TC-PNs the ideal tools to clean modern/contemporary masterpieces such as paints by Jackson Pollock, Pablo Picasso, Roy Lichtenstein, and others.^124−126^

**Figure 6:**
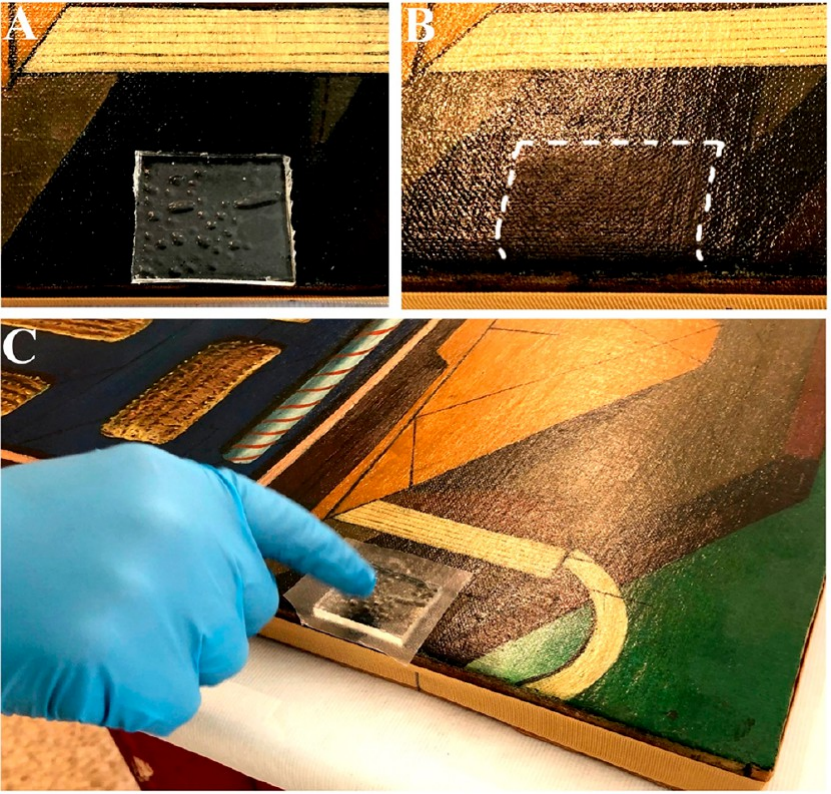
Removal of an aged varnish from the surface of a modern art masterpiece of metaphysical painting by Giorgio De Chirico (Le Doux Après-midi; oil painting, 1916; Peggy Guggenheim Collection, Venice; Solomon R. Guggenheim Foundation, New York), using a castor oil-based organogel loaded with a cleaning solvent. (A) Solvent-loaded organogel applied on the varnished surface. (B) Grazing light image showing the shiny varnish and the area where it was safely and completely removed by the organogel, bringing back the original matte colored surface. (C) Organogel covered with a plastic film to limit solvent evaporation during the application (from 30 s to 1 min). Courtesy of Piero Baglioni.

While the pHEMA/PVP semi-IPNs and TC-PNs are being increasingly adopted as standards in cleaning interventions, new research efforts are now focusing on the design of “green” gel formulations based on biomaterials.^127^ For instance, starch can partially replace PVA in the TC-PNs,^128^ while castor oil^129^ or polyhydroxybutyrate^130^ has been recently used to build organogels, complementary tools to hydrogels when conservators wish to control the use of organic solvents rather than aqueous systems. Figure 6 shows an example where a castor oil-based organogel, loaded with a cleaning solvent, is used to selectively remove an aged varnish from a modern art masterpiece of metaphysical painting. Other recent promising approaches include the cross-linking of chitosan, l-cysteine, and itaconic anhydride to yield networks able to uptake metallic ions,^131^ thiol–ene photopolymerization,^132^ or hydrogels obtained from renewable and biodegradable sources via the so-called Michael addition reaction.^133,134^

## Conclusions

The conservation of cultural heritage is a challenging task that must be addressed to transfer invaluable assets to future generations and to grant resilience and socioeconomic benefits to our society. This overview shows how colloids and soft matter have generated, in the past decades, a significant breakthrough in conservation science, moving from classical solution and polymer chemistry to systems or processes that exploit interfacial physicochemical phenomena, using, whenever possible, materials with higher compatibility with the original components of works of art. As a result, numerous valuable and time-effective solutions have been produced and made available to conservators worldwide, tackling the consolidation, protection, and cleaning of works of art. Systems like dispersions of nanoparticles, films, or hybrid organic–inorganic matrices, microemulsions, and hydro- or organogels have been explored and applied to conservation case studies, spanning from the Classic age and Renaissance to the complex and highly challenging restoration of modern and contemporary art. Both the remedial and preventive conservation of collections have been targeted, producing a wide palette of solutions; substrates and supporting systems for advanced diagnostics have also been developed. These advancements have been widely disseminated in the scientific and citizen community, as shown by the growing number of publications in the literature that, starting from pioneering works, report on advanced solutions for cultural heritage preservation. In this sense, the contribution of journals dedicated to colloids and soft matter, such as *Langmuir*, has been central in promoting a paradigm shift in material and methodologies for art preservation.

However, despite the remarkable achievements and the setting of new standards in conservation, the task is still far from concluded. Current and future challenges are focused on the development of fully sustainable technologies to cope with the demands of the Green Deal. Therefore, the use of sustainable secondary raw materials, natural substances, and eco-friendly nanomaterials has become a central topic in the design of new conservation tools.^135^ Inspiration in designing novel sustainable materials with enhanced properties can also come from ancient civilizations and cultures, where nanomaterials and interfacial phenomena were exploited based on empirical knowledge. Examples include archeological or paleontological materials that survived to recent times,^136^ or the enhanced properties of Maya plasters (that mixed biomacromolecules in calcium carbonate biominerals)^137^ and Roman cement.^138^ With today’s awareness and scientific understanding of colloidal and interfacial processes, the time thus is ripe for a new, sustainable breakthrough in the preservation of works of art.
